# Crystal structure of a 4-thiouridine synthetase–RNA complex reveals specificity of tRNA U8 modification

**DOI:** 10.1093/nar/gku249

**Published:** 2014-04-05

**Authors:** Piotr Neumann, Kristina Lakomek, Peter-Thomas Naumann, Whitney M. Erwin, Charles T. Lauhon, Ralf Ficner

**Affiliations:** 1 Department of Molecular Structural Biology, Institute of Microbiology and Genetics, GZMB, University of Göttingen, 37077 Göttingen, Germany; 2 School of Pharmacy, University of Wisconsin, Madison, WI 53705, USA

## Abstract

In prokaryotes and archaea transfer ribonucleic acid (tRNA) stability as well as cellular UV protection relies on the post-transcriptional modification of uracil at position 8 (U8) of tRNAs by the 4-thiouridine synthetase ThiI. Here, we report three crystal structures of ThiI from *Thermotoga maritima* in complex with a truncated tRNA. The RNA is mainly bound by the N-terminal ferredoxin-like domain (NFLD) and the THUMP domain of one subunit within the ThiI homo-dimer thereby positioning the U8 close to the catalytic center in the pyrophosphatase domain of the other subunit. The recognition of the 3’-CCA end by the THUMP domain yields a molecular ruler defining the specificity for U8 thiolation. This first structure of a THUMP/NFLD-RNA complex might serve as paradigm for the RNA recognition by THUMP domains of other proteins. The ternary ThiI–RNA–ATP complex shows no significant structural changes due to adenosine triphosphate (ATP) binding, but two different states of active site loops are observed independent of the nucleotide loading state. Thereby conformational changes of the active site are coupled with conformational changes of the bound RNA. The ThiI–RNA complex structures indicate that full-length tRNA has to adopt a non-canonical conformation upon binding to ThiI.

## INTRODUCTION

In all domains of life ribonucleic acid (RNA) maturation includes the post-transcriptional modification of nucleosides (1–3). The widest variety and largest number of modified nucleosides have been identified in transfer RNAs (tRNAs). Among them are 16 different thio-nucleotides including the 4-thiouridine (s^4^U), which is ubiquitously found and always located at position 8 in the loop region connecting the acceptor stem and the D stem of eubacterial and archaeal tRNAs. s^4^U8 does not only stabilize the fold of the tRNA (4,5), but additionally plays a central role in bacterial UV protection as it acts as a sensor for near-UV radiation (6–9).

The biosynthesis of s^4^U8 is catalyzed by 4-thiouridine synthetase (ThiI) (10), which consists at least of three domains: an N-terminal ferredoxin-like domain (NFLD), a THUMP domain and a pyrophosphatase (PPase) domain. In γ-proteobacteria, such as *Escherichia coli*, and in species of the archaeon *Thermoplasma*, ThiI contains a fourth, C-terminal rhodanese-like domain (RLD) (11). The crystal structures of ThiI from *Bacillus anthracis* (ThiI*_Ba_*) and of a putative ThiI ortholog from the archaeon *Pyrococcus horikoshii* have been solved (12,13). Based on the ThiI*_Ba_* structure and the results of biochemical studies, a functional assignment of the three common domains (NFLD, THUMP, PPase) has been proposed (12,14). While the NFLD and the THUMP domain form the major bipartite RNA binding module recognizing the tRNA acceptor stem region, the PPase domain binds adenosine triphosphate (ATP) by the PP-loop motif SGGxDS and contains the catalytic center (12,15).

The catalytic mechanism of 4-thiouridine synthesis is not yet understood in detail, though a multi-step reaction mechanism was proposed for ThiI from *E. coli* (ThiI*_Ec_*) requiring the cysteine desulfurase IscS (16–19). The sulfur transfer from cysteine to ThiI involves two consecutive formations of an internal persulfide at a cysteine residue of firstly IscS and secondly of the RLD of ThiI (18). After activation of U8 by adenylation, catalyzed by ThiI's PPase domain, two reaction pathways were considered: either the cysteine persulfide of the RLD performs a direct nucleophilic attack on the adenylated U8 or the terminal sulfur of the persulfide is liberated as hydrogen sulfide, which serves as a nucleophile to displace the activated oxygen of the uracil base (11,19). In both scenarios, finally adenosine monophosphate (AMP) and s^4^U8-tRNA are released, and a disulfide bond between the cysteine of the RLD and a cysteine of the PPase domain are formed (20). For the regeneration of the active enzyme this disulfide bond has to be reduced, hence, *in vitro* ThiI catalyzes only a single turnover in the absence of a reducing agent (20,21). In contrast, ThiI orthologs lacking the RLD seem to apply a different mechanism as recently demonstrated for ThiI orthologs from *Bacillus subtilis* (22) and *Methanococcus maripaludis* (23) involving the sulfurtransferase NifZ or IscS, respectively. Here, the persulfide is generated on a cysteine of the PPase domain, and finally a disulfide is formed with another cysteine of the PPase active site. This mechanism is reminiscent of that of the 2-thiouridine synthetase MnmA, which modifies U34 in the anticodon of some tRNAs (24).

Since the target U8 is buried in the core of canonical L-form tRNA, conformational changes have to occur in order to make it accessible for ThiI (14), but so far it has been unknown how U8 is specifically recognized. A truncated tRNA consisting of 39 nucleotides (TPHE39A) was previously shown to serve as minimal substrate for s^4^U8 synthesis (25). It corresponds to the acceptor and T-stems of substrate tRNA^Phe^ from *E. coli* as nucleotides G1-A7 and U29-A39 of TPHE39A match G1-A7 and U66-C72 of the acceptor-stem of tRNA^Phe^, and C15-G28 of TPHE39A are equivalent to C49-G65 in the T-stem of tRNA^Phe^ (Supplementary Figure S1). The bulge (U8-C14) connecting both stems of TPHE39A is composed of the target U8, the adjacent A9 and the variable loop sequence G10-C14, which is equivalent to G44-C48 of the full-length tRNA^Phe^. Surprisingly, even though the 3D structure of TPHE39A differs in the T-stem and T-loop in comparison to full-length tRNA (14), it can be specifically modified by ThiI (25).

In order to gain insight into the RNA binding mode of ThiI and to understand the structural basis of ThiI's specificity for both tRNA and the target U8, we determined the crystal structure of ThiI from *Thermotoga maritima* (ThiI*_Tm_*) in complex with the truncated tRNA^Phe^ (TPHE39A) from *E. coli*. With regard to the crystal structure of ThiI*_Ba_*, the structure of each domain of ThiI*_Tm_* remains unchanged upon RNA binding, but their relative orientation to each other changes considerably. The tRNA acceptor stem and the crucial single-stranded ACCA 3’-end form multiple interactions with the NFLD and THUMP domain of one monomer, respectively. This binding mode enables correct positioning of U8 close to the active site in the PPase domain of the other subunit within the homo-dimeric ThiI. The complex structure also suggests that the conformation of full-length substrate tRNA has to largely deviate from the canonical L-shape for productive binding.

## MATERIALS AND METHODS

### Crystallization

The overexpression and purification of ThiI*_Tm_*, and the preparation and crystallization of the ThiI*_Tm_*–RNA complex, has previously been described (26). Briefly, 1 μl of complex (ThiI–RNA complex 10 mg/ml, 20 mM Tris/HCl, pH 7.5, 150 mM (NH_4_)_2_SO_4_) was mixed with 3 μl of reservoir (2 M sodium formate, 100 mM sodium citrate, pH 4.6, 2 mM DTT). Single crystals were obtained at 20°C after 1 week in sitting drops by vapor diffusion both in the presence of ATP (2 mM) and MgCl_2_ (2 mM) or in the absence of ATP and MgCl_2_. For data collection, crystals were transferred into cryo buffer (2 M sodium formate, 100 mM sodium citrate pH 4.6, 30% (v/v) glycerol).

### X-ray diffraction data collection

The X-ray diffraction data of two crystals (ThiI*_Tm_*–RNA and ThiI*_Tm_*–RNA–ATP**)** have been collected at the beamline X11 of the EMBL outstation Hamburg at DESY, Germany, at 100 K and a wavelength of 0.9780 Å and 0.8140 Å, respectively. The diffraction images of the ThiI*_Tm_*–RNA–ATP complex have been processed with the XDS package (27,28) and of the ThiI*_Tm_*–RNA complex with MOSFLM/SCALA (29,30). X-ray diffraction data statistics are summarized in Table 1.

**Table 1:** Data collection and refinement statistics

	Dehydrated crystal	Non-dehydrated crystal	Non-dehydrated crystal
	ThiI*_Tm_*–RNA(FMS)	ThiI*_Tm_*–RNA	ThiI*_Tm_*–RNA–ATP
**Data collection**			
Beamline	BL14.1, BESSY	X11, DESY/EMBL	X11, DESY/EMBL
	Berlin, Germany	Hamburg, Germany	Hamburg, Germany
Detector	MARCCD 165mm	MARCCD 165mm	MARCCD 165mm
Wavelength (Å)	1.008500	0.978000	0.814000
Space group	P2_1_2_1_2_1_	P2_1_2_1_2_1_	P2_1_2_1_2_1_
Cell dimensions			
*a*, *b*, *c* (Å)	100.65, 110.33, 119.31	103.28, 113.55, 132.15	103.17, 113.22, 133.18
Resolution (Å)	37.18–2.85	57.07–3.50	44.45–3.42
	(2.95–2.85)^a^	(3.69–3.50)^a^	(3.52–3.42)^a^
*R*_merge_^b^ (%)	8.5 (62.6)	8.9 (61.1)	9.8 (67.0)
*I*/σ(*I)*	16.42 (2.06)	9.9 (2.1)	14.8 (2.5)
Wilson B (Å^2^)	57.5	102.5	76.5
Completeness (%)	96.4 (81.4)	99.3 (99.9)	97.9 (84.7)
Refl. total/unique	198702/30547	85440/20061	76901/21271
Redundancy	6.2 (2.7)	4.3 (4.1)	3.6 (3.3)
Software used for data processing	XDS/XSCALE	MOSFLM/SCALA	XDS/XSCALE

**Refinement**			
Resolution (Å)	37.18–2.85	29.75–3.50	29.39–3.42
	(2.95–2.85)	(3.68–3.50)	(3.63–3.42)
No. reflections	30363	19940	21154
*R*_work_ (%)	18.61 (27.36)	23.02 (30.12)	23.04 (38.58)
*R*_free_^c^ (%)	22.83 (31.53)	26.86 (33.81)	27.38 (41.23)
No. atoms	7976	7860	7924
Protein	6231	6198	6198
RNA	1666	1662	1662
Ligand/ion	4	-	64
Water	75	-	-
*B*-factors (Å^2^)	83.3	159.2	103.1
Protein	66.2	148.6	91.3
RNA	148.5	198.6	147.3
Ligand/ion	140.7	-	94.8
Water	55.2	-	-
Solvent content (%)	57.8	64.0	64.1
R.m.s. deviations			
Bond lengths (Å)	0.012	0.009	0.010
Bond angles (°)	0.927	0.979	0.968
Ramachandran plot, favored/disallowed (%)	95.7/0.5	90.2/3.3	94.3/1.17
Coordinate error (Å)^d^	0.26	0.48	0.60
PDB code	4KR6	4KR9	4KR7

^a^Values in parentheses are for the highest-resolution shell.

^b^*R*_merge_ = Σ_hkl_ Σ_i_ |I_i_(hkl) - < I(hkl)>|/Σ_hkl_ Σ_i_ I_i_(hkl).

^c^*R*_free_ factor calculated for 5% randomly chosen reflections not included in the refinement.

^d^Maximum-likelihood based coordinate error estimated by the refinement program (PHENIX).

### Controlled crystal dehydration

In order to improve the resolution of the X-ray diffraction data a controlled crystal dehydration experiment was performed. The crystal was mounted in a LithoLoop (Molecular Dimensions Ltd, Soham, UK) and placed into the head of the Free Mounting System (FMS; Proteros Biostructures GmbH, Martinsried, Germany) in an adjustable and reproducible stream of humidified gas, which allows a controlled change of relative humidity (31). At the beginning the relative humidity was set to 91.8% of that of the mother liquor (99.9%). After applying the humidity gradient, the diffraction quality of the crystal was monitored by collecting a series of diffraction images. The greatest improvement of the diffraction properties of the crystal was achieved at a relative humidity of 83% causing a reduction of the unit cell volume by 15%. When the relative humidity was reduced below this value (the lowest 79.8%) the diffraction deteriorated, however returning to the optimal relative humidity of 83% restored the diffraction properties of the crystal. The crystal was flashed cooled after coating it in a thin film of perfluoropolyether oil and used for the further diffraction data collection using synchrotron radiation where it diffracted X-rays to the maximum resolution limit of 2.85 Å. Diffraction data were collected at the beamline BL14.1 at BESSY, Berlin, Germany (32), at 100 K and a wavelength of 1.0085 Å on a marccd 165 detector in rotation steps of 0.5°. The oscillation images have been processed using the XDS package (27,28).

### Structure determination of the ThiI*_Tm_*–RNA complex using a dehydrated crystal

The ThiI*_Tm_*–RNA complex crystallized in the space group P2_1_2_1_2_1_ (*a* = 100.65 Å, *b* = 110.33 Å, *c* = 119.31 Å, α = β = γ = 90°) with two protein and two minimal substrate RNA molecules in the asymmetric unit (solvent content of 68.7%). The data set collected from the crystal treated by controlled dehydration using the FMS was used in order to solve the phase problem by Molecular Replacement (MR) method and build the model. The known crystal structure of ThiI*_Ba_* (12) sharing 40% identity with ThiI*_Tm_* was used and the search was performed using PHASER (33). First the largest domain (residues 177–388) of the RNA-free structure of ThiI*_Ba_* with all non-conserved residues replaced by serine has been located. The two missing domains comprising the residues 1–77 and 78–163, respectively, were placed based on superposition of the ThiI*_Ba_* monomer and optimized by rigid body refinement using the program package CNS (34,35). The model was rebuilt in COOT (36) and refined with strong non-crystallographic symmetry (NCS) restraints using CNS. The NCS restraints were later relaxed for regions showing conformational differences between the two monomers. When the *R*-factors dropped below 27.5% (*R*_work_) and 30.3% (*R*_free_), the density for oligonucleotides became visible and allowed manual building of the two TPHE39A molecules. The model was validated against SIGMAA-weighted simulated annealing (SA) (difference) omit maps as calculated with CNS. The last refinement steps were performed using PHENIX (37,38) with eight translation, libration and screw motion (TLS) groups allowing modeling of anisotropic displacements of individual ThiI domains and two RNA molecules. The final model consisting of residues Met1-Glu388 and nucleotides 1–39 for both protein monomers and RNA molecules, 78 water molecules and four partially occupied mercury ions (derived from soaking the crystal with HgCl_2_ prior FMS treatment) was refined at the resolution of 2.85 Å to *R*_work_ and *R*_free_ factors of 18.5% and 22.7%, respectively. Stereochemical quality of the structure was assessed by MolProbity (39,40) (95.98% of the residues are located within the favored regions of the Ramachandran plot and 0.52% in the disallowed regions).

### Structure determination of ThiI*_Tm_*–RNA complexes at low resolution

The ThiI*_Tm_*–RNA–ATP complex structure has been solved by MR using PHASER and the high-resolution structure of ThiI*_Tm_*–RNA_FMS_ as the starting model. Several MR searches have been performed in order to localize the protein part using separate ThiI*_Tm_* monomers. The resulting solutions of symmetrical and asymmetrical ThiI dimers (monomer compositions AA, AB, BB, BA) have been scored according to the log-likelihood gain (LLG) values as calculated by PHASER. The protein model revealing the highest LLG score has been used as a partial solution for the search of tRNA molecules using truncated models of TPHE39A (3’-ACCA end and nucleotides forming the loop regions, U11 to C15 and A21 to C24, have been deleted). The structure was manually rebuilt and verified against SIGMAA-weighted SA omit maps, composite omit maps and difference maps using COOT. The structure was refined in CNS using strong NCS restraints between equivalent residues in the two monomers and weaker ones between acceptor and T stems of tRNA molecules. The NCS restraints were later relaxed for regions showing different conformations in each ThiI*_Tm_* monomer (N-termini, loop regions 11 to 22, 287 to 295, 333 to 350). For refinement, 5% of the reflections were randomly chosen and left out for cross-validation as the free *R*-factor. In order to reduce the risk of over-fitting, refinement was based on slow-cooling SA (torsion angle dynamics) combined with standard minimization and grouped *B*-factor refinement. Remaining non-interpreted electron density in the active site clearly exhibited the size and shape of a bound nucleotide triphosphate. After adding an ATP molecule to both catalytic centers, in each active site a difference peak of mFo-DFc electron density at a contour level of 3.5 sigma has been observed within 2.5–3.3 Å distances to three oxygens of the triphosphate moiety. Those peaks have been interpreted as Mg^2+^ ions as they were present in the crystallization buffer. Refinement of individual atomic displacement factors for the Mg^2+^ ions resulted in *B*-factors in the same range as those of the ATP molecules (refined as grouped *B*-factor). Non-ideal Mg^2+^–O distances could result from low resolution of the diffraction data and consequently larger positional error, however we can not rule out the possibility that the observed difference electron density peaks could correspond to a highly coordinated water molecules or a mixture of the two mentioned individuals.

The final protein model consists of residues 3–388 for each of the ThiI*_Tm_* monomers and two copies of TPHE39A (nucleotides 1–39). To model anisotropic displacements of the ThiI domains and tRNA molecules, we performed the final refinement step using PHENIX with eight TLS groups at 3.42 Å resolution resulting in *R*_work_ and *R*_free_ factors of 23.04% and 27.38%, respectively. The ATP-free ThiI*_Tm_*–RNA complex structure is isostructural with the ThiI*_Tm_*–RNA–ATP complex structure and has been refined using the same strategy at the resolution of 3.5 Å to the *R*_work_ and *R*_free_ factors of 23.02% and 26.86%, respectively.

### Structure analysis

The contact areas between the two ThiI molecules in the asymmetric unit were analyzed with the program PISA (41). The calculated complexation significance score amounted to 1.0, which is the maximal possible value, suggesting the existence of a stable homo-dimer. A *B*-factor sharpened SA omit electron density map of ATP was calculated with CNS (35). Figures were generated using PyMOL (The PyMOL Molecular Graphics System, Version 1.5.0.4 Schrödinger, LLC). Analysis of RNA secondary structures and 2D secondary structure plots were done with the program RNAview (42,43).

### Complementation of *E. coli ΔthiI* (DE3)

*E. coli ΔthiI* (DE3) cells were transformed with the pET15b plasmids encoding either wild-type ThiI*_Tm_*, or ThiI*_Tm_* C165S or ThiI*_Tm_* C344S. Overnight cultures (3 ml) were grown in lysogeny broth (LB) medium in the presence of 50 μg/ml ampicillin at 37ºC with shaking at 250 rpm and used to seed 50 ml cultures. When the cultures reached an OD_600_ = 0.6, expression was induced with isopropyl β-D-1-thiogalactopyranoside (IPTG) (1 mM) and the temperature was raised to 45ºC for the remainder of the overnight incubation period. Unfractionated tRNA was isolated, digested and analyzed using high pressure liquid chromatography (HPLC) at a wavelength of 330 nm as previously described (17). Samples from the ThiI*_Tm_* complementation were compared to a sample from the wt *E. coli* strain MC1061 grown overnight in LB at 37ºC.

### ThiI*_Tm_* DEAE filter disc activity assay

Reactions (50 μl) containing 50 mM Tris, pH 7.5, 5 mM Mg(OAc)_2_, 50 mM KCl, 4 mM ATP, 1 mM DTT, 10 μM tRNA substrate, 0.5 μg IscS*_Tm_* and 0.5 μg of wt or mutant ThiI*_Tm_* were mixed and initiated by the addition of 20 μM L-cysteine. The mixtures were incubated at 80ºC for 2 min. Aliquots (25 μl) were then removed and applied to DE81 filter discs (Whatman) and processed for scintillation counting as previously described (25).

### HPLC assay for AMP formation during ThiI*_Tm_* catalyzed s^4^U formation

Complete reaction mixtures (50 μl) contained 50 mM Tris, pH 7.5, 5 mM MgCl_2_, 50 mM KCl, 0.2 mM ATP, 1 mM DTT, 1 μg IscS*_Tm_*, 1 mg ThiI*_Tm_* and 20 μM TPHE39A (or tRNA^Phe^) RNA. After incubation for 10 min at 37°C, mixtures were analyzed by HPLC using a Supelco LC-18S column eluted with a 40 min gradient of 0–20% methanol in 20 mM potassium phosphate, pH 6.0, with UV detection at 260 nm.

## RESULTS

### Crystal structure determination

ThiI from the hyperthermophilic bacterium *T. maritima* (ThiI*_Tm_*) was crystallized with bound truncated tRNA^Phe^ (TPHE39A) from *E. coli* (26). ThiI*_Tm_* is capable to complement an *E. coli* Δ*thiI* strain by modifying U8 of *E. coli* tRNAs (Supplementary Figure S2), and the truncated tRNA^Phe^ TPHE39A from *E. coli* is *in vitro* an excellent substrate for ThiI*_Tm_*, showing similar levels of s^4^U modification as a full-length tRNA transcript (Supplementary Figure S3). The crystals usually exhibited poor diffraction properties, but optimization of the crystallization and crystal cryo-protection conditions followed by an extensive crystal screening yielded a few crystals diffracting to a resolution limit of about 3.5 Å. The diffraction quality of one ThiI*_Tm_*-TPHE39A crystal was further improved by controlled dehydration providing data to 2.85 Å resolution (ThiI*_Tm_*–RNA_FMS_), concomitantly leading to significant unit cell shrinkage (Table 1). The ThiI*_Tm_*–RNA_FMS_ complex structure could be solved by MR only when using separated domains of the RNA-free structure of ThiI*_Ba_* (12) as search models. After rebuilding the structures of the two ThiI molecules present in the asymmetric unit, two TPHE39A molecules were manually built based on difference electron density maps (Supplementary Figure S4), and the crystal structure of the ThiI*_Tm_*–RNA_FMS_ complex was refined at a resolution of 2.85 Å. Additionally, two ThiI*_Tm_*–RNA complex structures, both from non-dehydrated crystals, have been solved by MR using fragments of the ThiI*_Tm_*–RNA_FMS_ structure as search models. One of these complexes is ATP-free (ThiI*_Tm_*–RNA), while the other one represents an ATP-bound form (ThiI*_Tm_*–RNA–ATP). These structures were refined at a resolution of 3.50 Å and 3.42 Å, respectively, with reasonable stereochemistry and *R*-values (Table 1). All structures exhibit elevated values of the atomic displacement factors (*B*-factors) reflecting a high degree of crystal disorder most likely caused by the high flexibility of the ThiI–RNA complexes (see below), which correlates with the limited resolution and the Wilson *B*-factors of the corresponding diffraction data.

### Overall structure of the ThiI_*Tm*_–RNA complex

**Figure 1.**
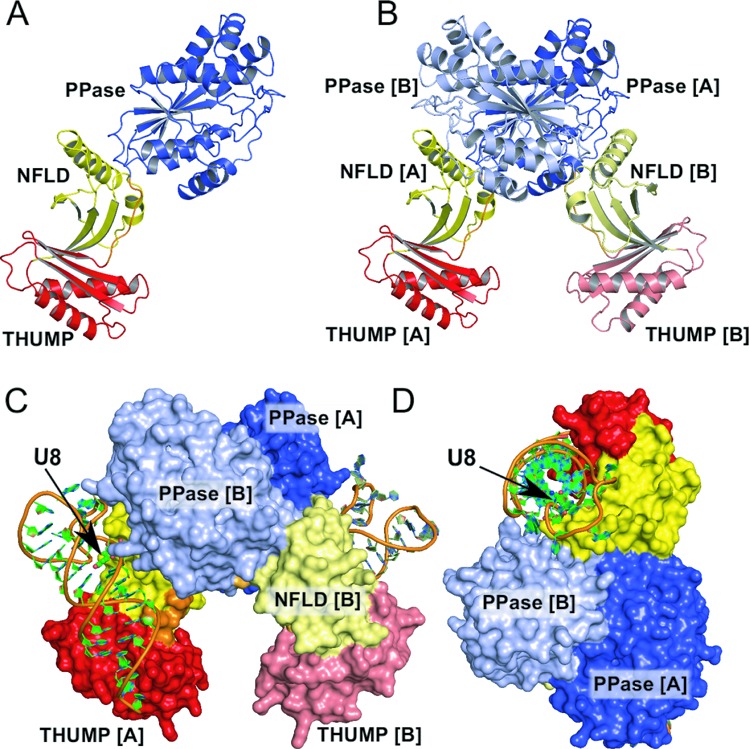
Structure of the ThiI*_Tm_*–RNA complex. (**A**) Ribbon representation of the ThiI*_Tm_* monomer consisting of a NFLD (yellow), a THUMP domain (red) and a pyrophosphatase (PPase) domain (blue). (**B**) Ribbon representation of the ThiI homo-dimer. The dimerization interface is mainly formed by the PPase domains. (**C**) The ThiI*_Tm_* homo-dimer binds two RNA molecules. The THUMP domain and the NFLD bind the acceptor stem, thereby positioning the U8 (position indicated by an arrow) close to the active site of the PPase domain of the other monomer. (**D**) View perpendicular to (C), showing how the three domains belonging to both monomers wrap around the RNA.

The ThiI*_Tm_* monomer has an elongated overall shape and comprises three structural domains: an N-terminal ferredoxin-like domain (NFLD: Met1-Lys72), followed by a THUMP domain (Gly73-Asp161) that is connected via a short flexible linker region (Arg162-Leu169) with the C-terminal PPase domain (Pro170-Glu388) (Figure 1A). In the 3D structure, the NFLD is located between the THUMP and PPase domains, hence, the linker connecting the THUMP and PPase domains crosses it. The structures of the ThiI*_Tm_* domains and their overall arrangement are similar to that of ThiI*_Ba_* (12), but the relative positions of the domains differ significantly (Supplementary Figure S5).

The asymmetric unit of the crystals contains two ThiI*_Tm_* protein molecules, each binding one RNA molecule. The size and properties of the contact surface between the two ThiI*_Tm_* molecules in the asymmetric unit indicate the presence of a physiologically relevant homo-dimer (Figure 1B). The dimer interface buries an area of about 3720 Å^2^, which corresponds to about 11% of each monomer's solvent accessible surface area (32,488 Å^2^). The interactions at the interface include 23 hydrogen bonds and 10 salt bridges, which are mainly formed by residues of the PPase domain and partially extend to the NFLD (three interactions), but exclude the THUMP domain. One hydrogen bond is formed between the backbone amide group of Gly168, which is part of the flexible linker, and the side chain of Asp319 located in the PPase domain of the other monomer. The dimer observed for the ThiI*_Tm_*–RNA complex corresponds to the dimer of ThiI*_Ba_* defined by crystallographic 2-fold symmetry axis. The superposition of ThiI*_Ba_* and ThiI*_Tm_* dimers unveiled that only the PPase domains represent the rigid and well superimposable part (r.m.s.d. of 1.4 Å for 301 Cα atoms).

Each of the two RNA molecules present in the crystal's asymmetric unit interacts with both subunits of the ThiI dimer (Figure 1C and D). The RNA is primarily bound by the NFLD and THUMP domain, while the bulge region harboring the U8 is located close to the active site of the PPase domain of the other subunit (Supplementary Table S1). The fact that the ThiI homo-dimer binds the RNA molecule by combining the catalytic PPase domain of one subunit with the NFLD and THUMP domain of the other subunit clearly demonstrates that the functional entity of ThiI is a homo-dimer, which can bind two RNA molecules simultaneously. Notably, the majority of the residues involved in RNA-binding are conserved among ThiI orthologs.

### Flexibility of the ThiI_*Tm*_ homo-dimer

**Figure 2.**
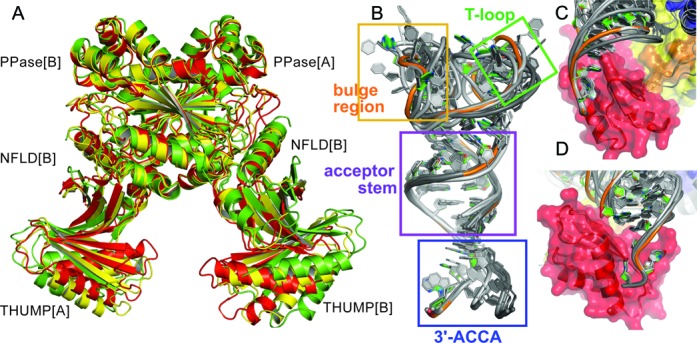
Flexibility of the ThiI*_Tm_*–RNA complex structure. (**A**) Structures of three ThiI dimers are compared by the best-fit superposition of their PPase domains highlighting the deviation from the symmetric ThiI*_Ba_* structure and the different relative orientations of the THUMP domains. Perfectly symmetric ThiI*_Ba_* is shown in green, the structure of ThiI*_Tm_* of the ThiI*_Tm_*–RNA complex is colored yellow and the structure of ThiI*_Tm_* obtained from the dehydrated crystals of the ThiI*_Tm_*–RNA complex is colored red. Due to the lack of significant conformational changes of the PPase domains and almost perfect superposition the ribbons are not clearly distinguishable for the individual PPase domains. (**B**) Superposition of the six RNA molecules present in the asymmetric units of the three crystals of ThiI*_Tm_*–RNA complexes indicating different flexibilities for the 3’-ACCA end (blue rectangular), T-loop (green rectangular) and the bulge region (orange rectangular). The acceptor stem (purple rectangular) and T-stem (not marked) are structurally very similar. (**C, D**) Two perpendicular views showing the recognition of the 3’-ACCA end by the THUMP domain. The interactions of the 3’-ACCA end with the THUMP domain are almost identical for all six ThiI*_Tm_*–RNA complexes and apparently independent of their different overall structures.

The comparison of the ThiI*_Ba_* and ThiI*_Tm_*–RNA structures reveals an asymmetry of the ThiI*_Tm_* homo-dimer due to different relative orientations of the NFLD and THUMP domains with respect to the PPase domains (Figure 2A). This flexibility of the protein correlates with differences in the conformation and orientation of the two TPHE39A molecules and non-identical RNA–protein interactions even within each homo-dimer. The homo-dimer's asymmetry was assessed by superimposing the PPase domains of ThiI*_Tm_* and ThiI*_Ba_* and then measuring the rotational angle and the magnitude of the translation vector required to obtain a perfect superposition of the individual NFLD and THUMP domains of the ThiI*_Tm_*–RNA complex structures onto the equivalent domains of the symmetrical ThiI*_Ba_* dimer (Supplementary Table S2). Interestingly, the observed asymmetry correlates well with the number of crystal and protein–RNA contacts and does not cause significant structural changes within the individual domains as indicated by negligible r.m.s.d. values obtained from domain-wise comparison. The largest differences are observed for the ThiI*_Tm_*–RNA_FMS_ structure, most likely caused by the crystal dehydration and unit cell shrinkage. The ThiI*_Tm_*–RNA and ThiI*_Tm_*–RNA–ATP structures show a similar, moderate degree of asymmetry indicating that ATP binding does not cause large conformational changes in the protein.

### RNA conformation

**Figure 3.**
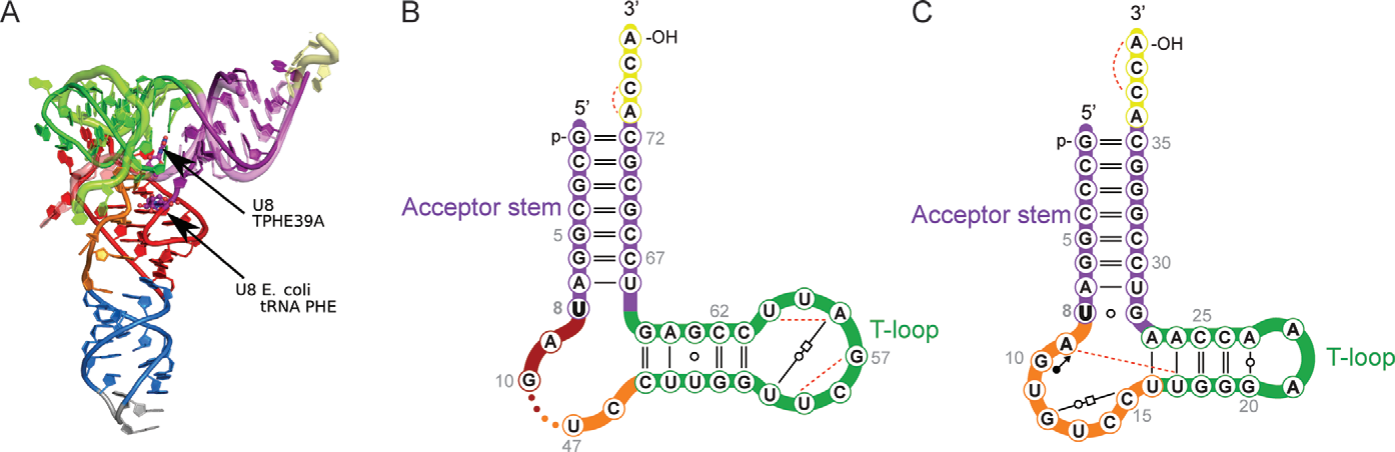
RNA structure. (**A**) Superposition of the crystal structure of unmodified tRNA^Phe^ from *E. coli* (PDB: 3L0U (44)) and the truncated tRNA^Phe^ (TPHE39A RNA) structure as observed in the complex structure with ThiI*_Tm_*. (**B, C**) Structure-based secondary structure representation of (C) TPHE39A RNA (chain X of PDB 4KR7) and (B) equivalent portion of tRNA^Phe^ (PDB: 3L0U) with all non-canonical interactions depicted as Leontis–Westhof symbols (43). The isolated open circles (○) denote GU wobble pairs. The pair of an open circle and an open square connected by a solid line represents *trans* Watson–Crick/Hoogsteen interactions between antiparallel strands, while *trans* Watson–Crick/Watson–Crick pairs between parallel strands are denoted by an open circle embedded within a solid line. The filled circle with an arrow (•→) symbolizes *cis* Watson–Crick/Sugar interactions between antiparallel strands. Dashed lines represent single hydrogen bonds that are neither formed by the Watson–Crick face nor edge-to-edge. The symbols ‘–’ and ‘=’ encode AU and GC *cis* Watson–Crick base pairs, respectively.

The conformation of the bound TPHE39A molecules is very similar to that of crystallized unbound TPHE39A (14) and basically resembles the corresponding regions of tRNA^Phe^ (44) (Figure 3, Supplementary Figure S1). The acceptor stem perfectly exhibits the architecture common to cytoplasmic tRNAs as the canonical Watson–Crick base pairs are maintained. The T-stem equivalent (C15-G28) is distorted compared to the canonical tRNA structure (C49-G65). However, none of the canonical *trans* Watson–Crick Hoogsteen (U8-A14, U54-A58), *trans* Watson–Crick ‘Levitt’ (G15-C48), *trans* Watson–Crick sugar-edge (G18-U55) and *cis* Watson–Crick (G19-C56) base pairs found in unmodified tRNA^Phe^ (44) or mature tRNA (45) structures can be formed due to the respective lack of one of these nucleotides. Interestingly, some of these missing interactions in TPHE39A are substituted by other interactions involving the same set of atoms. Unlike in tRNA^Phe^, U8 of TPHE39A forms a wobble base pair with G28 (Figure 3), and A9 of TPHE39A interacts with both G10 of the bulge and U17 within the T-stem leading to a compaction of the structure. The contact of U54 in tRNA^Phe^ is replaced by a *trans* Watson–Crick/Watson–Crick interaction of the corresponding G20 to A23. C15, the equivalent of C48, builds a *trans* Watson–Crick/Hoogsteen pair with G12 of the bulge in an antiparallel orientation.

Comparison of the six independent TPHE39A molecules of the three dimeric ThiI*_Tm_*–RNA complex structures identified significant conformational differences in the bulge region (A9-C15) and the T-loop (G20-A23), while the acceptor stem and T-stem structures are very similar (Figure 2B, Supplementary Table S3). These deviations correlate with the number of crystal contacts and elevated atomic *B* factors. In contrast, the 3’-ACCA moiety is not involved in any crystal contacts and reveals the lowest atomic *B* factors within the RNA. It is tightly bound by the THUMP domain with almost identical conformation and pattern of RNA–protein interactions in all ThiI*_Tm_–RNA* complexes (Figure 2C and D). Strikingly, the position of the 3’-ACCA with respect to the acceptor stem deviates between RNA molecules bound by different ThiI*_Tm_* monomers. These differences correlate with a rotation around the bonds connecting the 3’-ACCA end with the acceptor stem, namely the phosphate group of A36 and the ribose of C35. These observations suggest that variations in relative orientations of the RNA-binding domains can be compensated by the intrinsic flexibility of the RNA molecule in order to preserve proper placement of the acceptor stem and the bulge region harboring U8.

### Structural basis for the specificity of U8 thiolation

ThiI modifies U8 of all bacterial tRNAs, hence there is no RNA sequence motif determining the specificity for U8 thiolation. As previously demonstrated in biochemical studies, the single-stranded 3’-ACCA end of tRNA^Phe^ is essential for the C4-thiolation of U8 (25). The ThiI*_Tm_*–RNA crystal structures demonstrate that indeed the highly specific binding of the 3’-ACCA end by the THUMP domain generates a molecular ruler defining the length from the 3’-ACCA end to the site of modification. The 3’-ACCA end is bound in a groove formed by the β-strand Thr102-Lys109, the α-helix Val118-Asn132 and the loop Phe133-Asp144, here referred to as CCA-binding loop.

**Figure 4.**
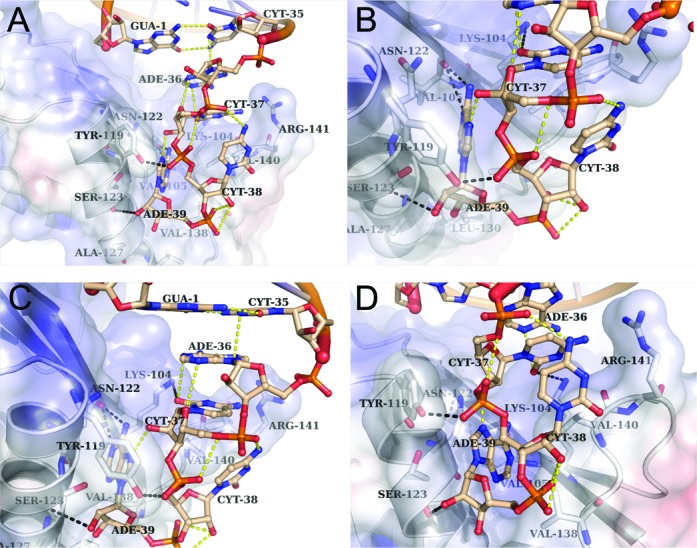
RNA-binding by the THUMP domain. (**A**) Hydrogen bonds between nucleotides of the single-stranded 3’-ACCA end and residues of the THUMP domain are shown as black dashed line, and hydrogen bonds within the RNA molecule are shown as yellow dashed line. (**B–D**) Three different enlarged views of (A) showing the interactions of nucleotides A39, C38, C37 and A36 with the THUMP domain.

The pattern of interactions between the 3’-ACCA moiety and the THUMP domain is very similar in all ThiI*_Tm_*–RNA structures (Figure 2C and D). The 3’-terminal A39 forms both polar contacts and hydrogen bonds with Ser123 and Val105, as well as with other nucleotides of the 3’-CCA end (Figure 4). In addition, several van-der-Waals contacts are made to residues of helix α4 (Asn122, Ser123, Gly126, Ala127), β-strand β6 (Lys104, Val105) and the CCA-binding loop (Val138). The next-to-last nucleotide, C38, forms one hydrogen bond to Tyr119, a polar interaction to Arg141 of the CCA-loop, and van-der-Waals contacts with the side chains of Val140 and Arg141. The conformation of C38 is further stabilized by interactions within the 3’-ACCA end. Like A39 and C38, C37 forms polar interactions with Lys104 and Val140, and within the 3’-ACCA. Since A36 functions as a flexible linker between the acceptor stem and the 3’-ACCA end, its interactions differ among the various ThiI–RNA complexes. As expected, the base of A36 forms stacking interactions with its adjacent nucleotides A37 and C35. In most of the complexes A36 is not bound by the protein, as it is placed more towards the solvent exposed part of the RNA, however, in one monomer of the ThiI*_Tm_*–RNA–ATP complex A36 forms hydrogen bonds with Lys104 and Gln106. The THUMP domain does not only bind the 3’-ACCA end but also the double-stranded acceptor stem by multiple contacts to nucleotides from G28 to A39 (for details see Supplementary Table S1). In addition to the THUMP domain the NFLD, which closely resembles the RNA recognition motif of ribosomal protein S6, also binds the RNA mainly by residues located in the loops Arg10-Lys19, and Trp44-Arg46, but there are also important contacts with residues of α-helix α1 (e.g. Arg21) and of β-strand β2 (e.g. Arg42).

### Catalytic center

**Figure 5.**
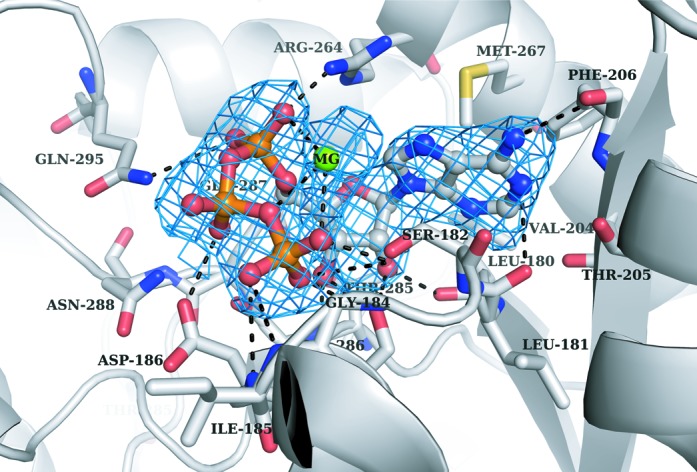
Active site of the ThiI*_Tm_* PPase domain. The *B*-factor sharpened (−80 Å^2^) SA omit mFo-DFc difference electron density map (colored blue) for the bound ATP molecule and the Mg^2+^ ion (MG) is contoured at 3 sigma. Residues of the PPase domain involved in ATP binding are shown as sticks, and hydrogen bonds or salt bridges are indicated as dashed lines.

The synthesis of 4-thiouridine requires the activation of U8 by adenylation, which leads to a covalent bond between O4 of U8 and the α-phosphate of ATP and to the release of pyrophosphate. We obtained crystal structures of ThiI*_Tm_*–RNA complexes in the ATP-free as well as ATP-bound form. Comparison of both structures depicts that ATP binding does not cause large conformational changes. The adenosine moiety of the ATP is bound in the active site of the PPase domain similarly to the ThiI*_Ba_*–AMP complex structure (12). Most residues involved in binding of the adenosine moiety are conserved between ThiI*_Tm_* and ThiI*_Ba_* (except Thr205 which is His208 in ThiI*_Ba_*) and have identical side chain conformations . The adenine ring is bound deeply in the active site and forms both polar and van-der-Waals interactions with ThiI*_Tm_* (Leu180, Leu181, Met267, Phe206, Ser182, Thr205, Val204)(Figure 5). The ribose makes several polar contacts with residues of the PP-loop (Leu180, Ser187) and of a loop at the dimer interface, here denoted as DI-loop (Gly286, Glu287). The phosphate moieties form a network of strong hydrogen bonds and polar interactions with the PP-loop (Ser182, Gly184, Ile185, Asp186, Ser187), residues of the DI-loop (Asn288, Gln291, Gln295) and Arg264. These interactions force the tri-phosphate of the bound ATP into a strongly kinked conformation, which should facilitate the nucleophilic attack of the flipped U8 on the α-phosphate. Remaining difference electron density at the ATP molecule was interpreted as Mg^2+^ ion located between the α- and γ-phosphate groups (Figure 5).

**Figure 6.**
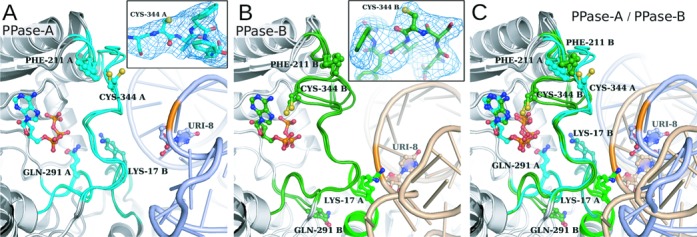
Two states of the active site loops within the dimeric ThiI*_Tm_*–RNA–ATP and ThiI*_Tm_*–RNA complexes. (**A, B**) Overlay of active sites with or without bound ATP are shown for the two ThiI*_Tm_* monomers present in the asymmetric unit. The active site loops are colored cyan for monomer A and green for monomer B. The inlets show the SA mFo-DFc difference omit maps contoured at 4 sigma (colored blue) of the active site loop containing the catalytic Cys344. Residues 340–348 were omitted for electron density map calculation. (**C**) The superposition of four ThiI*_Tm_* monomers shows large movement of the active site loop containing the catalytic cysteine (Cys344) leading from an open to a closed state. This loop rearrangement is accompanied by conformational changes of three other loops flanking the active site and correlates with the different positions and conformations of the bound RNA molecules.

The cysteine residue 344 within the PPase domain of ThiI*_Ec_* was shown to be essential for catalysis (19). Mutation of the corresponding Cys344 to serine in ThiI*_Tm_* leads to a complete loss of activity (Supplementary Figures S2 and S3). Notably, the active site loop carrying this catalytic cysteine is fully disordered in the ThiI*_Ba_*–AMP complex structure, whereas it is traceable in the ThiI*_Tm_*–RNA and ThiI*_Tm_*-ATP–RNA structures (Figure 6). While no conformational changes are induced upon ATP binding, the binding of RNA seems to force the active site loops into defined positions. Remarkably, different conformations are observed for four loops flanking the active site, when comparing the two active sites within the ThiI*_Tm_*–RNA–ATP and ThiI*_Tm_*–RNA complexes, respectively (Figure 6). They concern the NFLD loop Ser12–Arg21 (bottom loop), and the loops Pro210–Ser213, Ile289–Leu297 and Lys338–Asn351 (upper loop) of the PPase domain. In the ThiI*_Tm_*-ATP–RNA structure both active sites contain ATP, but the relative position of the RNA-bulge harboring the U8 with respect to the active site is quite different. Thus, the conformation of these active site loops and the position of the RNA substrate are directly correlated. Thereby conformational changes of the bound RNA are coupled with conformational changes of the active site. These structural rearrangements mainly concern the bottom (Ser12–Arg21) and upper (Lys338–Asn351) active site loops that comprise Lys17 and Lys19 or the catalytic Cys 344, respectively.

In case of the RNA molecule bound to the NFLD-B and THUMP-B, the bulge region is positioned further away from the PPase-A domain and makes only two interactions with the active site residue Lys350-A (Figure 6A). The NFLD-B loop (Ser12–Arg21) folds towards the loop Ile289–Leu297 of the PPase-A domain resulting in direct interactions. The side chain of Lys17-B points towards the active site of the PPase-A domain, while that of Gln291-A contacts the ATP phosphates (Figure 6A). The upper active site loop Ser336-A–Phe347-A has an extended conformation with the catalytic Cys344-A exposed to the solvent resulting in a very wide and open active site of PPase-A. In contrast, all three active site loops of the PPase-B domain exhibit different conformations (Figure 6B). The RNA is positioned closer to the PPase-B domain, so that its bulge region makes 17 contacts with the active site residues. The vicinity of the bulge region causes conformational changes in the NFLD-A loop. As a result, Lys17-A changes its position and is placed close to the RNA molecule so that its side chain interacts with the A9 phosphate. The loop Ile289-B–Leu297-B changes its conformation so that Gln291-B is located at the outer part of the active site with its side chain pointing towards the elongated α-helix of the NFLD-A. The active site loop of PPase-B folds towards the active site and partially covers it. Thus Cys344-B becomes buried and gets closer to the ATP, but still leaving the α-phosphate of the ATP accessible for the attack by U8 (Supplementary Figure S6).

## DISCUSSION

ThiI exhibits a structural modularity that combines a conserved catalytic domain with additional domains achieving specific substrate recognition, a strategy also applied by other RNA-modifying enzymes (46,47). A central role for the RNA recognition by ThiI plays the THUMP domain, which was initially identified *in silico* as putative RNA-binding domain (48). However, the mode of RNA binding by a THUMP domain remained elusive, as no structure of a THUMP–RNA complex has been known so far. The ThiI*_Tm_*–RNA complex structures unravel how the THUMP domain binds both the single-stranded 3’-ACCA end and the double-stranded acceptor stem. The strict dependence of ThiI activity on the 3’-ACCA end obviously guarantees that *in vivo* only correctly processed tRNAs are modified, as e.g. a blunt end tRNA reduces s^4^U8 synthesis to below 0.1% (25).

The structure of ThiI's THUMP domain closely resembles the ones found in pseudouridine synthase Pus10 (49), tRNA cytidine deaminase CDAT8 (50), and the RNA methyltransferases RlmKL (51), Trm14/TrmN (52) and RlmM (53). The residues of the ThiI*_Tm_* THUMP domain involved in RNA-binding are mainly conserved among all THUMP domains, while other solvent exposed areas of the ThiI*_Tm_* THUMP domain are rather variable (Supplementary Figure S7). Interestingly, with exception of Pus10 all of these enzymes contain also a NFLD that forms together with the THUMP domain a functional entity similar to that of ThiI. This applies especially for the tRNA-modifying enzyme CDAT8, which catalyzes a C-to-U editing of archaeal tRNAs at position 8 (50). The tRNA is expected to be recognized by the dimeric CDAT8 in an identical manner by the structurally conserved combination of NFLD and THUMP, thereby positioning C8 in the active site of the deaminase domain belonging to the other subunit of the homo-dimer (50). In case of the monomeric Trm14/TrmN, which methylates the guanine at position 6 of tRNA^Phe^, a ThiI-like binding of the tRNA by the THUMP/NFLD places G6 in proximity to the AdoMet molecule bound to the methyltransferases domain (52). However, the THUMP domain is not always accompanied by a NFLD, as seen in the pseudouridine synthase Pus10 (49), hence the redefinition of the THUMP domain by including the NFLD, as done by the SCOP database, appears to be misleading. Superposition of the ThiI and Pus10 THUMP domains reveals that Pus10 could bind the 3’-ACCA end like ThiI, but that the 5’ end of the tRNA would clash with the helical extension of the Pus10 THUMP domain. Hence, conformational rearrangements of the RNA and/or protein are expected for the Pus10-tRNA complex, e.g. a rotation of the 3’-ACCA end relative to the acceptor stem.

The crystal structures of the ThiI*_Tm_*–RNA complexes described here demonstrate that only dimeric ThiI is able to position U8 at the catalytic center of the PPase domain, and that the 3′-CCA end common to all tRNAs serves as the ‘reference point’ to measure the distance to the site of modification. An acceptor stem shortened by a single base pair prevents s^4^U8 synthesis, which is consistent with the structural data and appears to be physiologically reasonable as all bacterial tRNAs contain exactly seven base pairs in the acceptor stem. Interestingly, homo-dimerization as a prerequisite for catalysis has also been observed for some other RNA-modifying enzymes such as the pseudouridine synthase TruA (54) or CDAT8 (50).

RNA-complex structures of other tRNA-modifying enzymes revealed that the substrate tRNA is either bound primarily as a rigid body or it is remodeled significantly thereby increasing the affinity and/or enabling access to an otherwise buried target base. Based on these tRNA conformations required for catalysis tRNA-modifying enzymes have been divided into two families (55). Interestingly, a model of ThiI with full-length L-shape tRNA obtained by superimposing the acceptor stems of tRNA^Phe^ onto the ThiI-bound TPHE39A results in serious clashes between the PPase domain and the tRNA D- and anticodon stems (Supplementary Figure S8). Therefore, full-length substrate tRNAs are expected to undergo large conformational rearrangements upon binding to ThiI. This conclusion is supported by circular dichroism (CD) spectroscopic studies indicating conformational changes to occur in tRNA^Phe^ when it binds to ThiI (14). The non-standard λ-form of tRNA observed in complex with an archaeal transglycosylase (56) can be ruled out to be suitable for s^4^U8 synthesis, since it would also clash with ThiI's catalytic domain. Strikingly, the electrostatic surface potential of ThiI*_Tm_* reveals positively charged areas as putative binding sites for the D- and anticodon arms of full-length tRNA (Supplementary Figure S8B).

Although the overall structure of the three domains of the tripartite ThiI*_Tm_* and ThiI*_Ba_* orthologs is quite similar, significant rearrangements of the individual domains relative to each other can occur. They seem to represent an essential inherent feature of ThiI to accommodate productive substrate binding and target U8 recognition. Structural changes of loops around the active site in the PPase domain result in two main shapes of its rim. The ATP binding pocket is either rather shallow—probably preventing U8 to flip into it—or larger and wider making it accessible for ATP and U8 binding (Supplementary Figure S6). Interestingly, the information about the conformation of the bound RNA seems to be transferred to the ATP binding pocket. This signaling cascade employs a set of loops flanking the active site that originate on one side from the NFLD of one monomer or on the opposite side from the catalytic PPase domain. These loops undergo coinciding rearrangements and place the catalytically essential Cys344 of the active site loop close to the bound ATP when the RNA has a proper position and conformation for U8 flipping, thus facilitating specific s^4^U8 formation. However, the crystal structures do not show a flipped out U8 bound in the catalytic center or even the adenylated U8 in case of the ATP complex. Probably the flip of U8 and the adenylation reaction only occur in the presence of IscS bound to the ThiI–RNA complex, which would be consistent with biochemical studies (unpublished). Such an IscS-induced mechanism appears reasonable as the adenylated U8 could be attacked by the sulfide immediately after the flip and the activating adenylation reaction, thereby preventing a wasteful hydrolysis of the U8-AMP intermediate. To understand the underlying mechanism to greater extent, structural information on the ThiI–RNA–IscS complex and the U8-adenylated ThiI-bound RNA coordinated at the active site is needed.

The reaction mechanism of 4-thiouridine synthetase appears to show striking variability depending on the organism. In ThiI*_Ec_* the Cys456 of the RLD forms an essential persulfide which was suggested to either directly attack the adenylated U8 or to serve as a source for the generation of hydrogensulfide anion (HS^−^) which then performs the nucleophilic attack (19). In contrast to the RLD-containing ThiI*_Ec_*, which utilizes the sulfurtransferase IscS, the RLD-deficient ThiI from *Bacillus subitilis* (ThiI*_Bs_*) strictly depends on the sulfurtransferase NifZ, which transfers the sulfhydryl group onto a not yet identified cysteine of ThiI*_Bs_* (22). *In vitro*, ThiI and NifZ are sufficient to catalyze s^4^U8 synthesis, hence no additional RLD-protein is required. Recent studies on the RLD-deficient ThiI from *M. maripaludis* showed as well that in this methanogenic archaeon s^4^U8 synthesis does not require the RLD and that a conserved CXXC motif within the PPase active site forms the persulfide and disulfide. This mechanism is reminiscent of 2-thiouridine synthetase (MnmA), which exclusively modifies U34 in the anticodon loop of some tRNAs. In the crystal structure of MnmA*_Ec_* a disulfide bond between two active site cysteines (Cys102, Cys199) was observed (24).

However, for ThiI*_Tm_* the mechanism has to be unique as it contains only one cysteine in the active site (Cys344). There is only one additional cysteine residue (Cys165) present in ThiI*_Tm_* located in the loop linking NFLD and THUMP domain. However, Cys165 plays no role, as the C165S mutant of ThiI*_Tm_* is as active as wild-type protein both *in vivo* and *in vitro* (Supplementary Figures S2 and S3), which is consistent with the structure as Cys165 is much too distant from the active site (Supplementary Figures S6C and S6D). Since the reaction requires the formation of a disulfide bond, it is tempting to speculate that it is formed between Cys344 and the persulfide carrying Cys of the IscS sulfurtransferase. The formation of AMP during *in vitro* thiolation of TPHE39A by ThiI*_Tm_* indicates the adenylation of the substrate (Supplementary Figure S9), however, the covalent bond between the α-phosphate and U8 was not formed prior to crystallization of the ThiI-ATP–RNA complex as it was observed e.g. in one of the crystal structures of the 2-thiouridine synthetase MnmA (24). Hence, the obtained crystal structure of the ThiI-ATP–RNA complex represents an initial inactive state. The mechanism of ThiI*_Tm_* activation remains yet unclear, but an involvement of IscS in ThiI*_Tm_* activation appears to be a plausible option.

## ACCESSION NUMBERS

The atomic coordinates and structure factors have been deposited in the Protein Data Bank, www.rcsb.org (PDB ID code 4KR6, 4KR7, 4KR9).

## SUPPLEMENTARY DATA

Supplementary Data are available at NAR Online.

SUPPLEMENTARY DATA
