# Supplementary material for: Crystal structure of a 4-thiouridine synthetase–RNA complex reveals specificity of tRNA U8 modification
Source: Nucleic Acids Res. 2014 Apr 5;42(10):6673–85. doi: 10.1093/nar/gku249 (PMC4041423; doi:10.1093/nar/gku249)
Supplement: SUPPLEMENTARY DATA [file supp_42_10_6673__index.html]

Crystal structure of a 4-thiouridine synthetase–RNA complex reveals specificity of tRNA U8 modification — SUPPLEMENTARY DATA 

# Crystal structure of a 4-thiouridine synthetase–RNA complex reveals specificity of tRNA U8 modification

## SUPPLEMENTARY DATA

**Files in this Data Supplement:**

- SUPPLEMENTARY DATA
