# Supplementary material for: Crystal structure of a 4-thiouridine synthetase–RNA complex reveals specificity of tRNA U8 modification
Source: Nucleic Acids Res. 2014 Apr 5;42(10):6673–85. doi: 10.1093/nar/gku249 (PMC4041423; doi:10.1093/nar/gku249)
Supplement: SUPPLEMENTARY DATA [file supp_gku249_nar-00050-r-2014-File008.pdf]

Supplementary Information

Crystal structure of a 4-thiouridine synthetase - RNA complex reveals specificity of tRNA U8 modification

Piotr Neumann, Kristina Lakomek, Peter-Thomas Naumann, Whitney M. Erwin, Charles T. Lauhon and Ralf Ficner

Table S1
Protein - RNA contacts

**ThiI$_{Tm}$-RNA**		**ThiI$_{Tm}$-RNA-ATP**		**ThiI$_{Tm}$-RNA$_{FMS}$**	
RNA M	RNA X	RNA M	RNA X	RNA M	RNA X
NFLD:		NFLD:		NFLD:	
Lys 17 - Uri 8<br>- Ade 9<br>Glu 18 - **Ade 9**<br>- Gua 10<br>Arg 42 - **Cyt 15**	Lys 19 - **Uri 16**	Gly 15 - **Ade 7**<br>Ile 14 - Ade 7<br>Lys 17 - Uri 8<br>**- Ade 9**<br>Gly 18 - **Ade 9**<br>**- Gua 10**<br>Lys 22 - **Gua 12**		Asn 20 - **Cyt 14**<br>Arg 21 - **Cyt 15**<br>Lys 19 - Cyt 15<br>Lys 17 - **Cyt 15**<br>Arg 21 - Cyt 15<br>- **Uri 16**<br>Arg 42 - **Uri 17**<br>Lys 22 - Uri 17<br>Ser 12 - Uri 29	Glu 13 - **Ade 7**<br>- **Uri 8**<br>Arg 42 - **Cyt 14**<br>Glu 26 - **Cyt 14**<br>Glu 25 - Cyt 15<br>Glu 13 - **Cyt 15**<br>Glu 25 - **Cyt 15**<br>Arg 42 - **Cyt 15**<br>- **Uri 16**<br>Gly 45 - **Uri 29**<br>Trp 44 - Uri 29<br>Arg 46 - **Cyt 30**<br>Ser 12 - **Cyt 30**<br>Tyr 11 - Cyt 30<br>Asn 69 - **Cyt 31**<br>Ser 12 - **Cyt 31**
THUMP:		THUMP:		THUMP:	
Val 118 - Gua 1<br>Lys 108 - **Cyt 30**<br>Arg 152 - **Cyt 30**<br>Val 140 - **Cyt 37**<br>Tyr 119 - **Cyt 38**<br>Val 105 - **Ade 39**<br>Ser 123 - Ade 39<br>Leu 130 - Ade 39	Gln 106 - Gua 1<br>Lys 104 - **Cyt 37**<br>Val 140 - **Cyt 37**<br>Tyr 119 - **Cyt 38**<br>Val 140 – Cyt 38<br>Arg 141 - **Cyt 38**<br>Asn 122 - **Ade 39**<br>Ser 123 - **Ade 39**<br>Ala 127 - **Ade 39**<br>Leu 130 - Ade 39	Val 118 - Gua 1<br>Tyr 111 - **Gua 28**<br>Lys 108 - **Cyt 30**<br>Arg 152 - **Cyt 30**<br>Lys 104 - Cyt 37<br>Val 140 - Cyt 37<br>Arg 141 - **Cyt 37**<br>Tyr 119 - **Cyt 38**<br>Val 105 - **Ade 39**<br>Ala 127 - Ade 39<br>Leu 130 - Ade 39	Lys 104 - **Cyt 37**<br>Val 140 - Cyt 37<br>Tyr 119 - **Cyt 38**<br>Val 105 - **Ade 39**<br>Ser 123 - **Ade 39**<br>Ala 127 - **Ade 39**<br>Leu 130 - Ade 39	Gln 106 - **Gua 1**<br>Lys 108 - **Gua 28**<br>- **Uri 29**<br>Val 140 - Cyt 37<br>Lys 104 - **Cyt 37**<br>Tyr 119 - Cyt 37<br>- **Cyt 38**<br>Arg 141 - Cyt 38<br>Val 105 - **Ade 39**<br>Ser 123 - **Ade 39**<br>Ala 127 - **Ade 39**	Lys 109 - Gua 1<br>Val 118 - Gua 1<br>Lys 108 - **Uri 29**<br>Arg 152 - **Uri 29**<br>Tyr 119 - **Ade 36**<br>Gln 106 - **Ade 36**<br>Lys 104 - **Ade 36**<br>- **Cyt 37**<br>Val 140 - **Cyt 37**<br>Tyr 119 - Cyt 37<br>Val 105 - **Ade 39**<br>Ser 123 - **Ade 39**
PPASE:		PPASE:		PPASE:	
Lys 338 - **Gua 6**<br>Asn 351 - **Uri 11**<br>- **Ade 23**		Asn 351 - **Uri 11**		Gln 341 - **Gua 6**<br>Asn 351 - **Uri 13**<br>Lys 257 - **Uri 13**<br>Asn 351 - **Cyt 14**<br>Thr 354 - **Cyt 14**<br>Ala 353 - Cyt 14<br>Lys 257 - **Cyt 14**<br>Pro 339 - Cyt 31<br>Tyr 333 - **Gua 32**	

Contacts for distances closer than 3.3 Å are listed separately for the two RNA chains of each structure. Each RNA molecule interacts with the NFLD and THUMP domains of one ThiI protomer and the PPase domain of the second one. The contacts are sorted according to the numbering of RNA nucleotides. Bold font indicates presence of at least one polar contact between involved residues/nucleotides.

Table S2
Degree of asymmetry of ThiI$_{Tm}$ homo-dimers.

	ThiI$_{Tm}$-RNA$_{FMS}$	ThiI$_{Tm}$-RNA	ThiI$_{Tm}$-RNA-ATP
PPase domain:			
r.m.s.d. between domains [Å]/Cα atoms	0.38 / 219	0.32 / 204	0.24 / 202
r.m.s.d. to ThiI$_{Ba}$ domains [Å]/Cα atoms	1.4 / 386	1.2 / 376	1.3 / 368
NFLD domain:			
A, diff. in orientation [°]/[Å];	7.4 / 3.7	6.7 / 2.6	7.0 / 2.9
B, diff. in orientation [°]/[Å];	13.3 / 6.8	9.7 / 5.1	9.3 / 4.9
rmsd. between domains [Å]/Cα atoms	0.42 / 60	0.28 / 63	0.3/ 63
rmsd to ThiI$_{Ba}$ domain [Å]/Cα atoms	1.3 / 62	1.4 / 59	1.3 / 59
THUMP domain:			
A, diff. in orientation [°]/[Å]	22.4 / 7.8	10.8 / 3.5	10.9 / 3.6
B, diff. in orientation [°]/[Å]	19.3 / 10.8	11.4 / 7.6	10.7 / 7.2
rmsd. between domains [Å]/Cα atoms	0.26 / 89	0.07 / 89	0.08 / 89
rmsd. to ThiI$_{Ba}$ domain [Å]/Cα atoms	1.2 / 89	1.2 / 91	1.2 / 91
No. of crystal contacts: monomer ***A*** / ***B***	210 / 124	61 / 51	45 / 34
A, protein-RNA interactions short/long	39 / 221	22 / 143	26 / 130
B, protein-RNA interactions short/long	31 / 211	10 / 81	9 / 98

R.m.s.d. values between the domains were calculated on all Cα positions. The difference in the number of crystal contacts formed by the two protomers (A and B) reach 40 %, 17 % and 25 % for ThiI$_{Tm}$-RNA$_{FMS}$, ThiI$_{Tm}$-RNA and ThiI$_{Tm}$-RNA-ATP structures, respectively. This allows distinguishing the two ThiI$_{Tm}$ monomers in the crystal lattice and gives the opportunity for unbiased comparison of different ThiI$_{Tm}$-RNA structures. The monomer assignment is also consistent with the differences in orientation of RNA molecules relative to the NFLD and THUMP domains of ThiI$_{Tm}$-RNA and ThiI$_{Tm}$-RNA-ATP structures.

Table S3
Flexibility of the TPHE39A RNA structure.

Nucleotide number => sequence	ThiI$_{Tm}$-RNA (FMS) chain X	ThiI$_{Tm}$-RNA (FMS) chain M	ThiI$_{Tm}$-RNA (non FMS) chain X	ThiI$_{Tm}$-RNA (non FMS) chain M	ThiI$_{Tm}$-RNA-ATP (non FMS) chain X	ThiI$_{Tm}$-RNA-ATP (non FMS) chain M
1_2 => G-C	-	-	stacked	stacked	-	-
1_35 => G-C	+/+ cis	+/+ cis	+/+ cis	+/+ cis	+/+ cis	+/+ cis
2_34 => C-G	+/+ cis	+/+ cis	+/+ cis	+/+ cis	+/+ cis	+/+ cis
2_35 => C-C	stacked	-	-	-	-	-
3_4 => C-G	-	stacked	stacked	stacked	-	-
3_33 => C-G	+/+ cis	+/+ cis	+/+ cis	+/+ cis	+/+ cis	+/+ cis
4_32 => C-G	+/+ cis	+/+ cis	+/+ cis	+/+ cis	+/+ cis	+/+ cis
4_33 => C-G	-	-	-	-	-	stacked
5_6 => G-G	-	stacked	-	-	stacked	-
5_31 => G-C	+/+ cis	+/+ cis	+/+ cis	+/+ cis	+/+ cis	+/+ cis
6_30 => G-C	+/+ cis	+/+ cis	+/+ cis	+/+ cis	+/+ cis	+/+ cis
7_8 => A-U	-	stacked	-	-	-	-
7_29 => A-U	-/- cis	-/- cis	W/W cis	W/W cis	-/- cis	-/- cis
7_30 => A-C	-	stacked	-	-	-	-
8_28 => U-G	W/W cis	W/W cis	W/W cis	W/W cis	W/W cis	W/W cis
8_15 => U-C	-	S/S cis	-	-	-	-
9_10 => A-G	S/W cis	-	-	-	S/W cis	-
9_11 => A-U	-	S/W cis	-	-	-	-
9_16 => A-U	-	H/H tran	-	-	-	-
9_17 => A-U	H/H tran	-	H/H tran	H/H tran	-	-
10_15 => G-C	-	-	W/W cis	-	-	-
12_14 => G-C	./? cis	-	-	-	-	-
12_15 => G-C	-	-	-	-	S/W cis	-
13_15 => U-C	-	-	-	S/W cis	-	S/W cis
15_16 => C-U	H/H cis	-	-	-	-	-
16_27 => U-A	-	W/W cis	-/- cis	-/- cis	-/- cis	-/- cis
16_17 => U-U	-	-	-	-	-	stacked
17_26 => U-A	-/- cis	-	-/- cis	-/- cis	-/- cis	-/- cis
17_18 => U-G	-	-	-	-	stacked	-
18_19 => G-G	S/H cis	-	-	-	-	
18_25 => G-C	+/+ cis	+/+ cis	+/+ cis	+/+ cis	+/+ cis	+/+ cis
19_20 => G-G	-	stacked	-	-	-	-
19_24 => G-C	W/W cis	-	+/+ cis	+/+ cis	+/+ cis	+/+ cis
20_23 => G-A	-	-	W/W tran	-	W/W tran	-
20_24 => G-C	-	-	-	+/+ cis	-	stacked
25_26 => C-A	stacked	-	-	-	-	-
28_29 => G-U	stacked	-	stacked	stacked	-	stacked
32_33 => G-G	stacked	-	-	-	-	-
34_35 => G-C	stacked	-	-	-	-	-
37_39 => C-A	-	-	-	-	S/W tran	-

Analysis of the six TPHE39A RNA structures by the RNAView program (42). Base pair interactions are named according to Leontis/Westhof nomenclature (56): "W/W cis" is cis Watson-Crick/Watson-Crick, "W/W tran" is trans Watson-Crick/Watson-Crick, "H/H tran" is trans Hoogsteen/ Hoogsteen, "S/S cis" is trans Hoogsteen/Sugar edge, "S/W tran" is trans Sugar edge/Watson-Crick, "S/W cis" is cis Sugar edge/Watson-Crick, "stacked" is base stacking interactions; "+/+ cis" and "-/- cis" denote standard canonical interactions. The "-" denotes lack of interactions between specified base pairs.

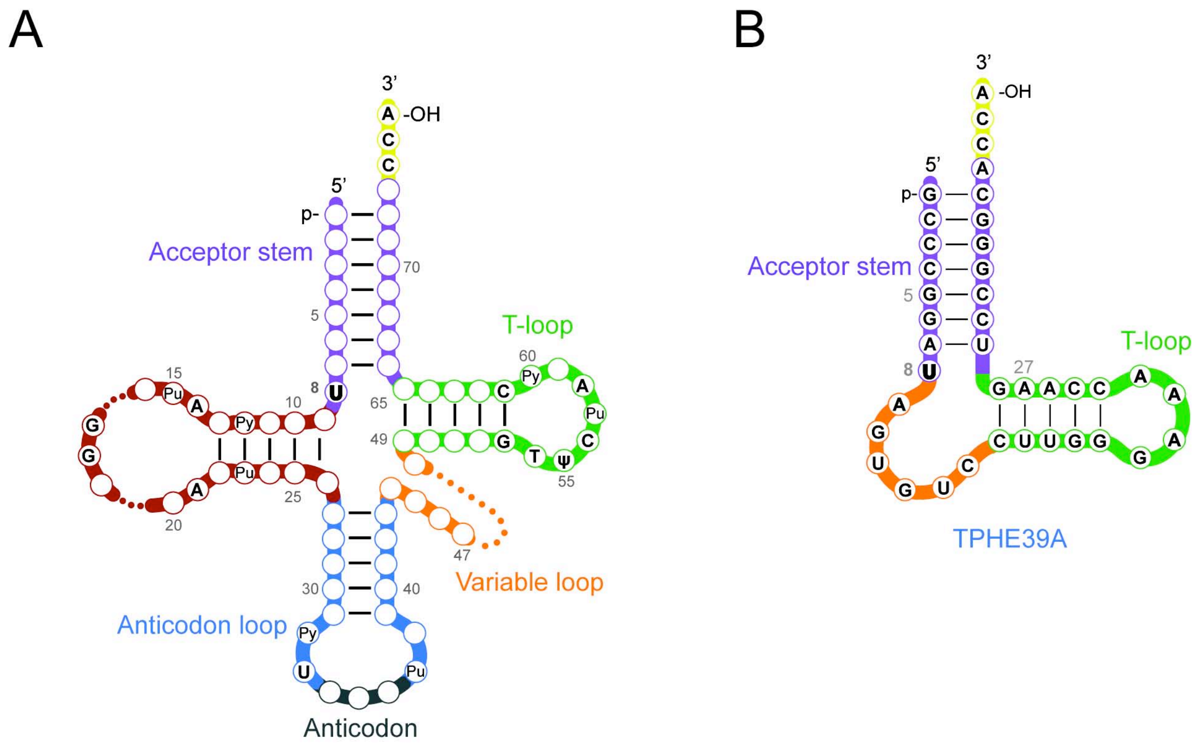

Figure S1: Idealized secondary structures of (A) bacterial tRNA and (B) truncated $tRNA^{Phe}$ from *E. coli* (TPHE39A), which is a minimal substrate for 4-thiouridine synthetase ThiI (25).

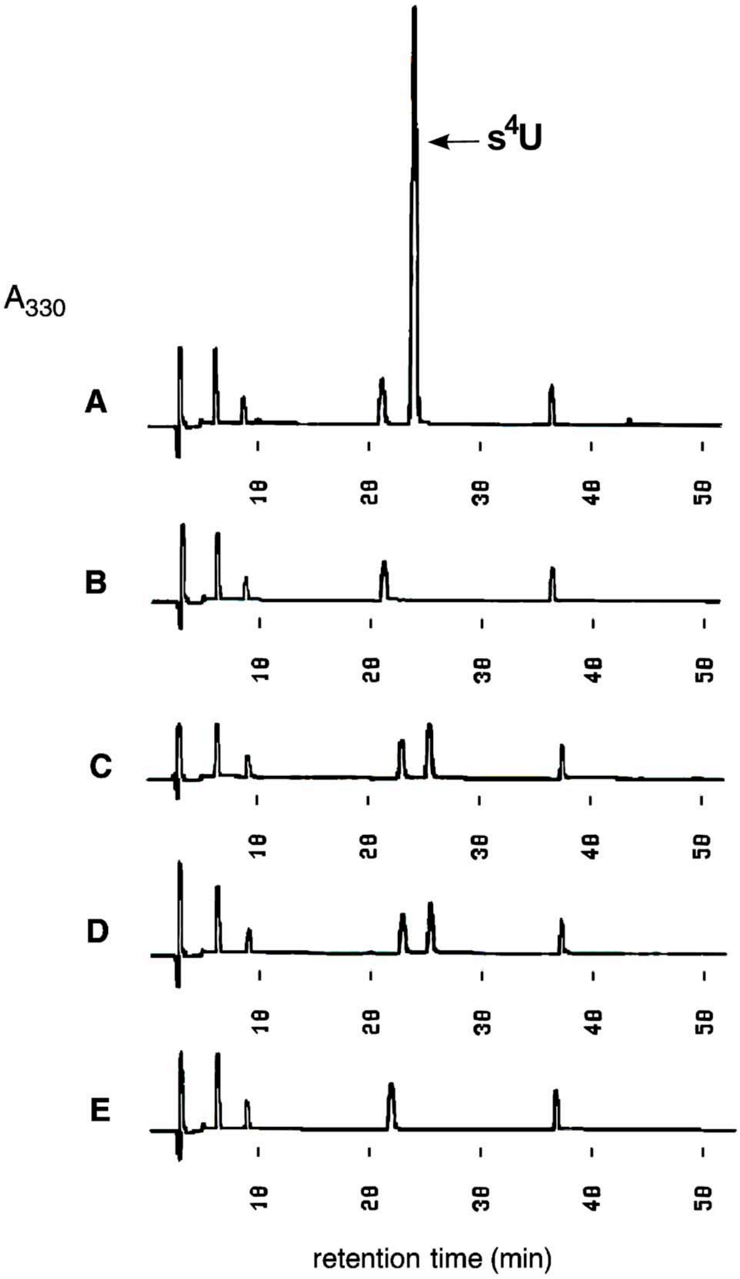

Figure S2: HPLC analysis (with UV detection at 330 nm) of tRNA isolated from a ΔthiI *E. coli* strain complemented with expression plasmids containing wildtype (wt) and mutant forms of the *thi* gene from *T. maritima*. The peak corresponding to s4U is indicated by an arrow (A) wt *E. coli* strain MC1061. (B) *E. coli* Δ*thiI* strain. (C) *E. coli* Δ*thiI* strain expressing wt $thiI_{Tm}$; (D) *E. coli* Δ*thiI* strain expressing the $thiI_{TM}$ C165S mutant; (E) *E. coli* Δ*thiI* strain expressing the $thiI_{Tm}$ C344S mutant.

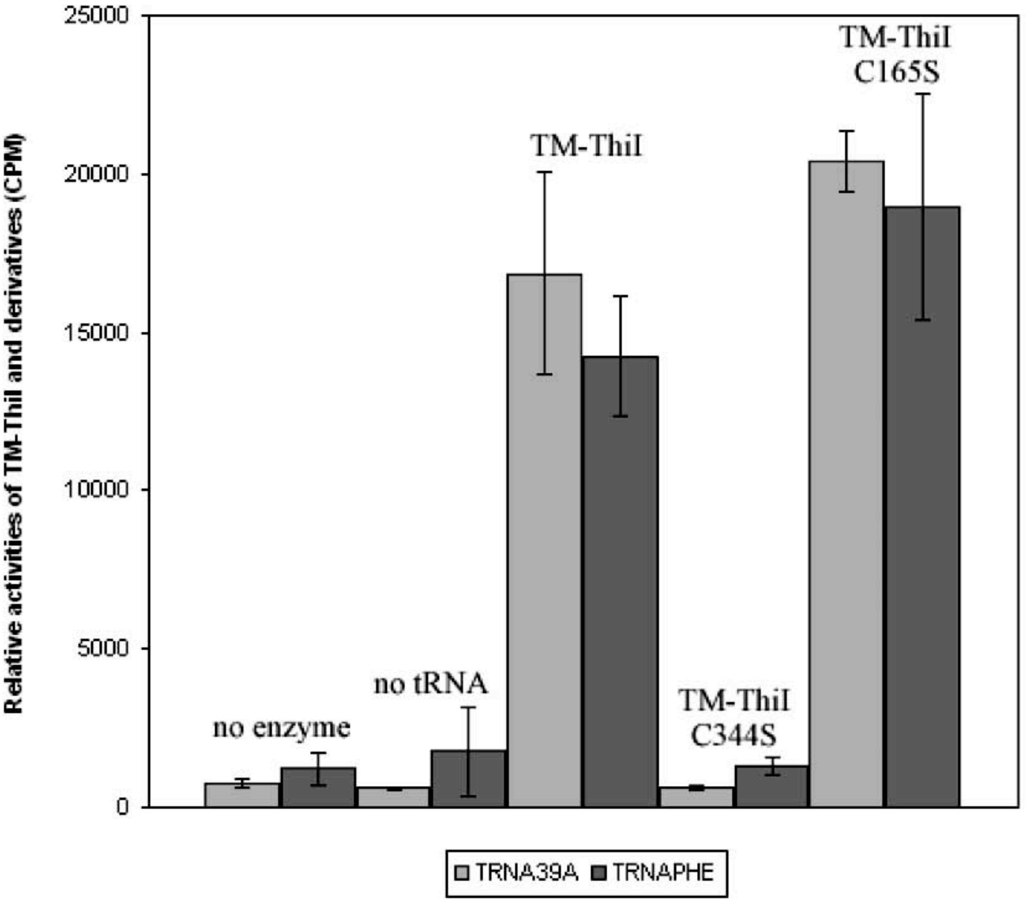

Figure S3: *In vitro* thiolation of RNA by ThiI$_{Tm}$. Relative activities of wildtype ThiI$_{Tm}$ (TM-ThiI), and mutated ThiI$_{Tm}$ (TM-ThiI C344S and TM-ThiI C165S) with tRNA^{Phe} or TPHE39A RNA transcript.

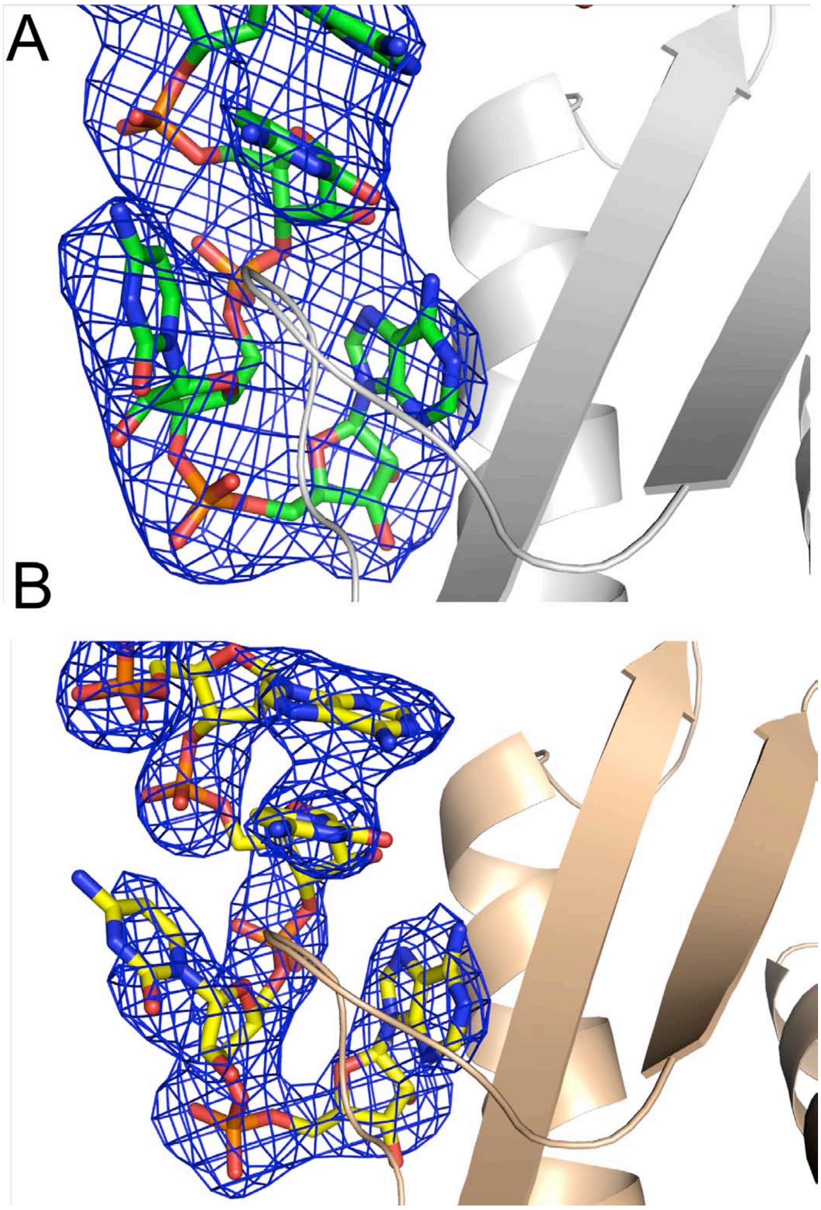

Figure S4: Simulated annealing (SA) omit mFo-DFc electron density map contoured at 3.0 sigma level showing the 3’-ACCA end of TPHE39A bound by ThiI$_{Tm}$. Nucleotides 35 - 39 were removed from the model for the calculation of the SA omit map. The selected RNA fragment is depicted as sticks and the fragment of the THUMP domain as cartoon representation. (A) Electron density at 3.4 Å resolution for the non-dehydrated crystal ThiI$_{Tm}$-RNA-ATP crystal, (B) Electron density at 2.85 Å resolution for the dehydrated crystal ThiI$_{Tm}$-RNA (FMS).

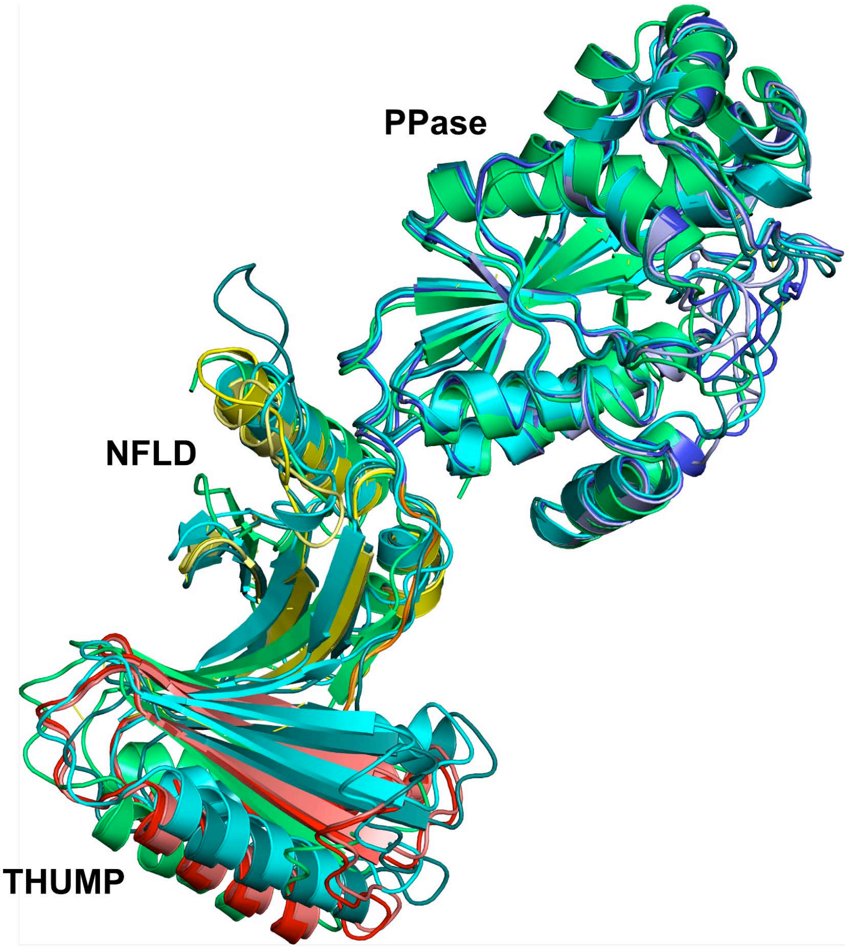

Figure S5: Superposition of individual monomers of the ThiI$_{Tm}$ structures and the ThiI$_{Ba}$ structure highlighting the conformational flexibility of ThiI as reflected by the different orientations of the THUMP domains relative to the PPase domains. ThiI$_{Ba}$ is shown in green, the structure of ThiI$_{Tm}$ of the ThiI$_{Tm}$-RNA complex is colored domain-wise (color code as in Figure 1B), and the structures of ThiI$_{Tm}$ monomers obtained from the dehydrated crystals of the ThiI$_{Tm}$-RNA complex are colored cyan and light blue. The superposition is based on the best-fit of the PPase domains. Due to partial perfect overlapping of the models the colors are not clearly distinguishable for the individual PPase domains.

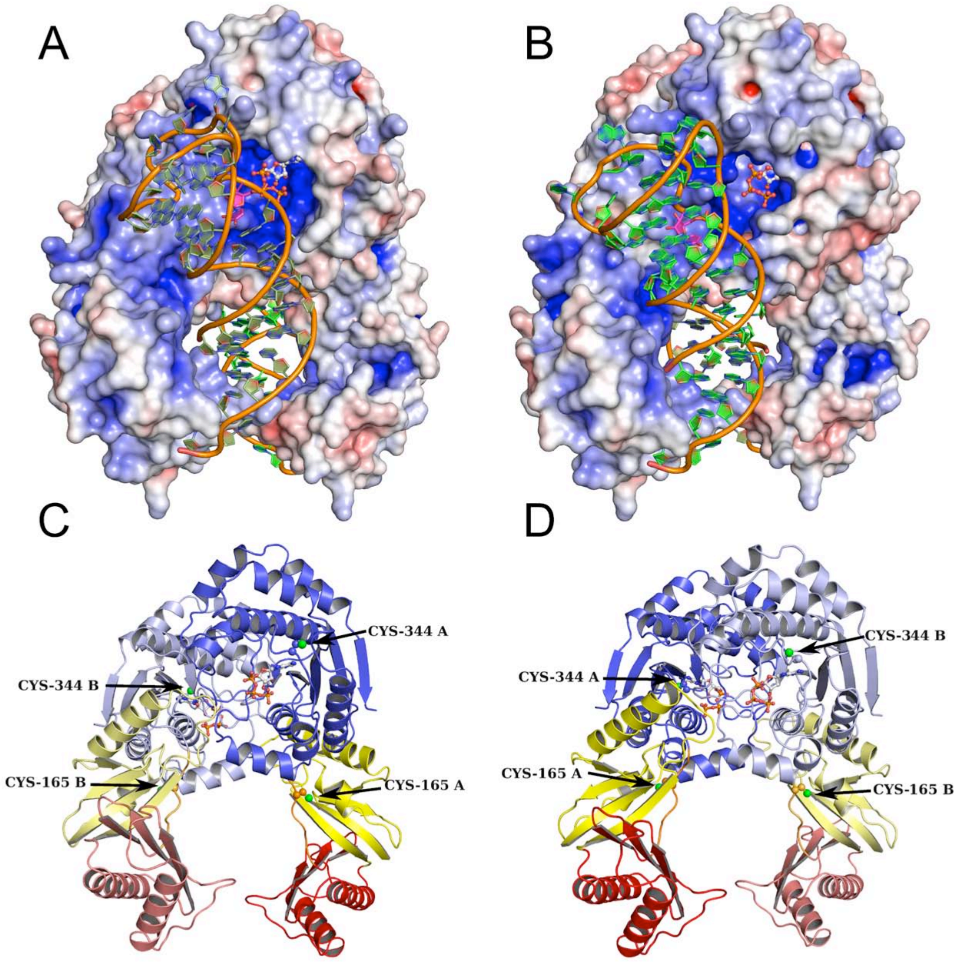

Figure S6. The different conformations of loops flanking the active site modulate the shape of the active site rim and cause a different accessibility of the ATP-binding pocket. (A,B) $ThiI_{Tm}$ is shown as surface representation with the bound RNA and ATP molecules. The electrostatic surface potential of $ThiI_{Tm}$ is rainbow colored from -5 kBT/e (red) to 5 kBT/e (blue). (A) The active site of PPase-A is in a more closed state with an almost buried adenosine moiety of the ATP. (B) The more open state of the active site of PPase-B might represent the conformation for initial binding of ATP. (C, D) Location of Cys165 and Cys344, the only cysteine residues of $ThiI_{Tm}$. The orientation of the ribbon representations in (C) and (D) corresponds to (A) and (B), respectively.

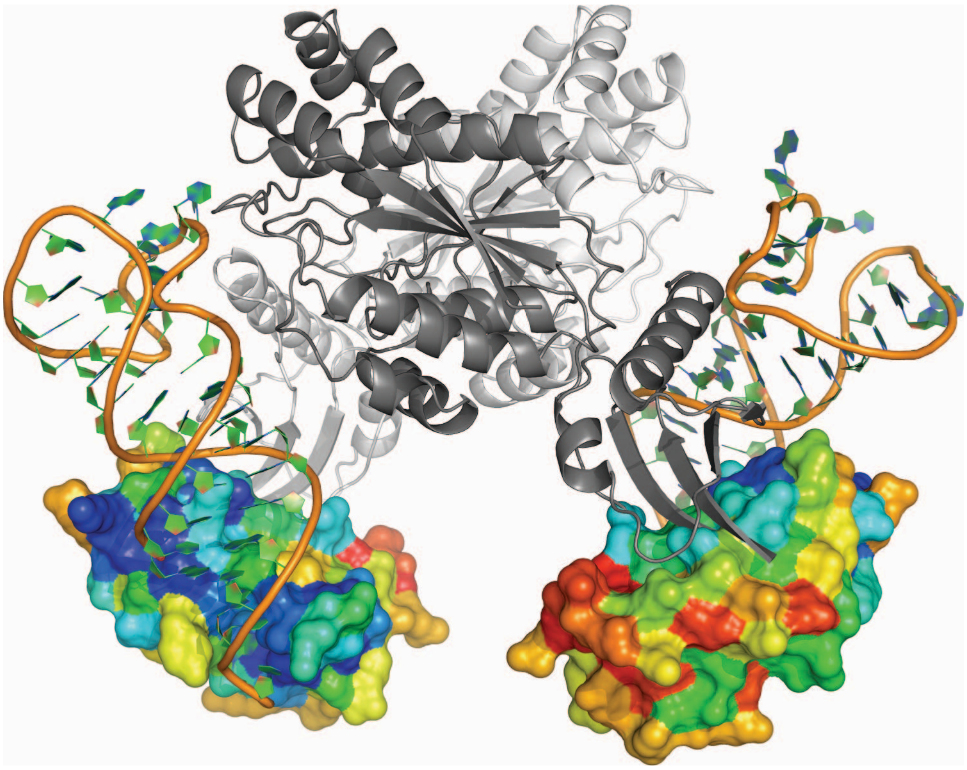

Figure S7: THUMP domain residues forming the RNA-binding surface are conserved among all known crystal structures of THUMP domains (blue surface color corresponds to identical residues, while red color are different residues. The rainbow coloring from blue to red indicates the degree of similarity).

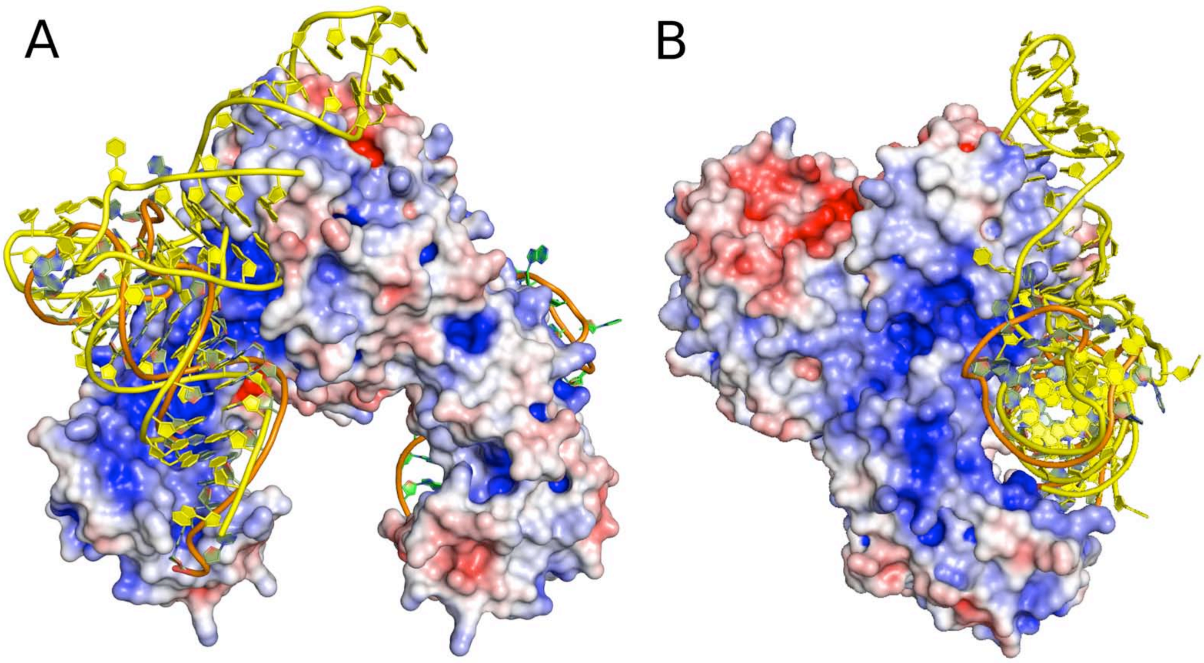

Figure S8: (A, B) The structure of $tRNA^{Phe}$ from *E. coli* (43) (PDB: 3L0U) superimposed onto the TPHE39A RNA molecule bound to $ThiI_{Tm}$ clearly suggests that the full-length tRNA molecule has to adopt a conformation significantly different from the canonical L-shape for productive binding to $ThiI_{Tm}$. The electrostatic surface potential of $ThiI_{Tm}$ is rainbow colored from -5 kBT/e (red) to +5 kBT/e (blue). (B) Positively charged surface areas (colored blue) are expected to be involved in binding of the D- and anticodon arms of full-length tRNA.

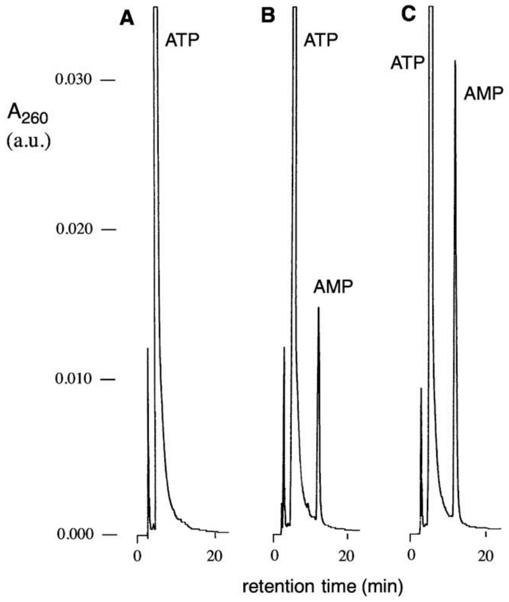

Figure S9. Formation of AMP during the *in vitro* s^4U modification of TPHE39A catalyzed by $ThiI_{Tm}$. (A) complete reaction mixture without RNA substrate; (B) complete reaction mixture with TPHE39A RNA; (C) co-injection of complete reaction mixture with AMP standard (1 nmol). The amount of AMP formation (840 pmol) corresponds to 84% of the amount of TPHE39A RNA
